# Computational Analysis of AMPK-Mediated Neuroprotection Suggests Acute Excitotoxic Bioenergetics and Glucose Dynamics Are Regulated by a Minimal Set of Critical Reactions

**DOI:** 10.1371/journal.pone.0148326

**Published:** 2016-02-03

**Authors:** Niamh M. C. Connolly, Beatrice D’Orsi, Naser Monsefi, Heinrich J. Huber, Jochen H. M. Prehn

**Affiliations:** 1 Centre for Systems Medicine, Department of Physiology & Medical Physics, Royal College of Surgeons in Ireland, Dublin 2, Ireland; 2 Centre for the Study of Neurological Disorders, Department of Physiology & Medical Physics, Royal College of Surgeons in Ireland, Dublin 2, Ireland; 3 Department of Cardiovascular Sciences, KU Leuven, 3000 Leuven, Belgium; University of Mississippi, UNITED STATES

## Abstract

Loss of ionic homeostasis during excitotoxic stress depletes ATP levels and activates the AMP-activated protein kinase (AMPK), re-establishing energy production by increased expression of glucose transporters on the plasma membrane. Here, we develop a computational model to test whether this AMPK-mediated glucose import can rapidly restore ATP levels following a transient excitotoxic insult. We demonstrate that a highly compact model, comprising a minimal set of critical reactions, can closely resemble the rapid dynamics and cell-to-cell heterogeneity of ATP levels and AMPK activity, as confirmed by single-cell fluorescence microscopy in rat primary cerebellar neurons exposed to glutamate excitotoxicity. The model further correctly predicted an excitotoxicity-induced elevation of intracellular glucose, and well resembled the delayed recovery and cell-to-cell heterogeneity of experimentally measured glucose dynamics. The model also predicted necrotic bioenergetic collapse and altered calcium dynamics following more severe excitotoxic insults. In conclusion, our data suggest that a minimal set of critical reactions may determine the acute bioenergetic response to transient excitotoxicity and that an AMPK-mediated increase in intracellular glucose may be sufficient to rapidly recover ATP levels following an excitotoxic insult.

## Introduction

Excitotoxicity, the excessive and pathological stimulation of neurons, is implicated in neuronal death in numerous neurological disorders including ischaemia, traumatic brain injury and neurodegenerative disease [1–3]. Although much is known about excitotoxicity, effective therapies are still lacking. Excitotoxic injury is mediated by glutamate receptor hyper-activation, severe calcium (Ca^2+^) influx and metabolic impairment, potentially culminating in neuronal death [4–6]. Neurons initially strive to extrude high and potentially toxic cytosolic Ca^2+^ by increasing the activity of plasma membrane ion pumps [3, 7], and by sequestering Ca^2+^ in the mitochondrial matrix, depolarising the mitochondrial membrane potential, Δψ_m_ [8]. Severe or prolonged glutamate exposure leads almost exclusively to neuronal necrosis, characterised by sustained Ca^2+^ deregulation and failure to restore adequate ATP levels [9–11]. Whenever excitotoxic stress is sufficiently transient or mild, however, neurons can recover equilibrium, characterised by Ca^2+^ homeostasis, stable ATP levels and intact ΔΨ_p_ and ΔΨ_m_ [6, 9, 12]. Despite recovery of homeostasis, however, some neurons nevertheless undergo delayed apoptosis [9, 13, 14].

We previously identified increased metabolism during the recovery phase that indicated the likelihood of neuronal survival [6, 11]. Survival was further associated with increased surface expression of the neuronal glucose transporter GLUT3, in a process dependent on the phosphorylation and activation of the AMP-activated protein kinase (AMPK) [12]. We hypothesised therefore that the AMPK-mediated increase in GLUT3 surface expression elevated glucose import and provided increased substrate for ATP production, restoration of energetic homeostasis and tolerance of the excitotoxic injury. Single-cell fluorescence measurements further demonstrated that ATP and AMPK activity promptly recovered to homeostasis following a transient insult, while the recovery of intracellular glucose was more delayed [15].

Here, we developed a computational model to test whether AMPK-mediated glucose import was sufficient to rapidly restore ATP following a transient excitotoxic insult. We aimed to focus on those processes that may be pathologically relevant for describing the biochemical cascade of altered bioenergetics, AMPK activation and GLUT3 surface expression triggered by cytosolic Ca^2+^ influx. We therefore employed a reductionist approach and here describe a minimal set of reactions to capture, from a top-down perspective, the essential mechanisms of the rapid and severe energetic perturbation during the glutamate excitotoxic response. Model inputs were calibrated and its predictions compared to previously published and *de novo* single-cell time-lapse fluorescence measurements in primary neurons from our lab [15]. We found that the acute bioenergetic response to transient excitotoxicity may be sufficiently described by a minimal set of critical reactions, and suggest that an AMPK-mediated intracellular glucose increase may critically contribute to rapidly recover ATP levels.

## Results

### A core computational model of calcium dynamics, energetic recovery and glucose import captures essential post-excitotoxic neuroprotective processes observed by single cell microscopy

**Fig 1:**
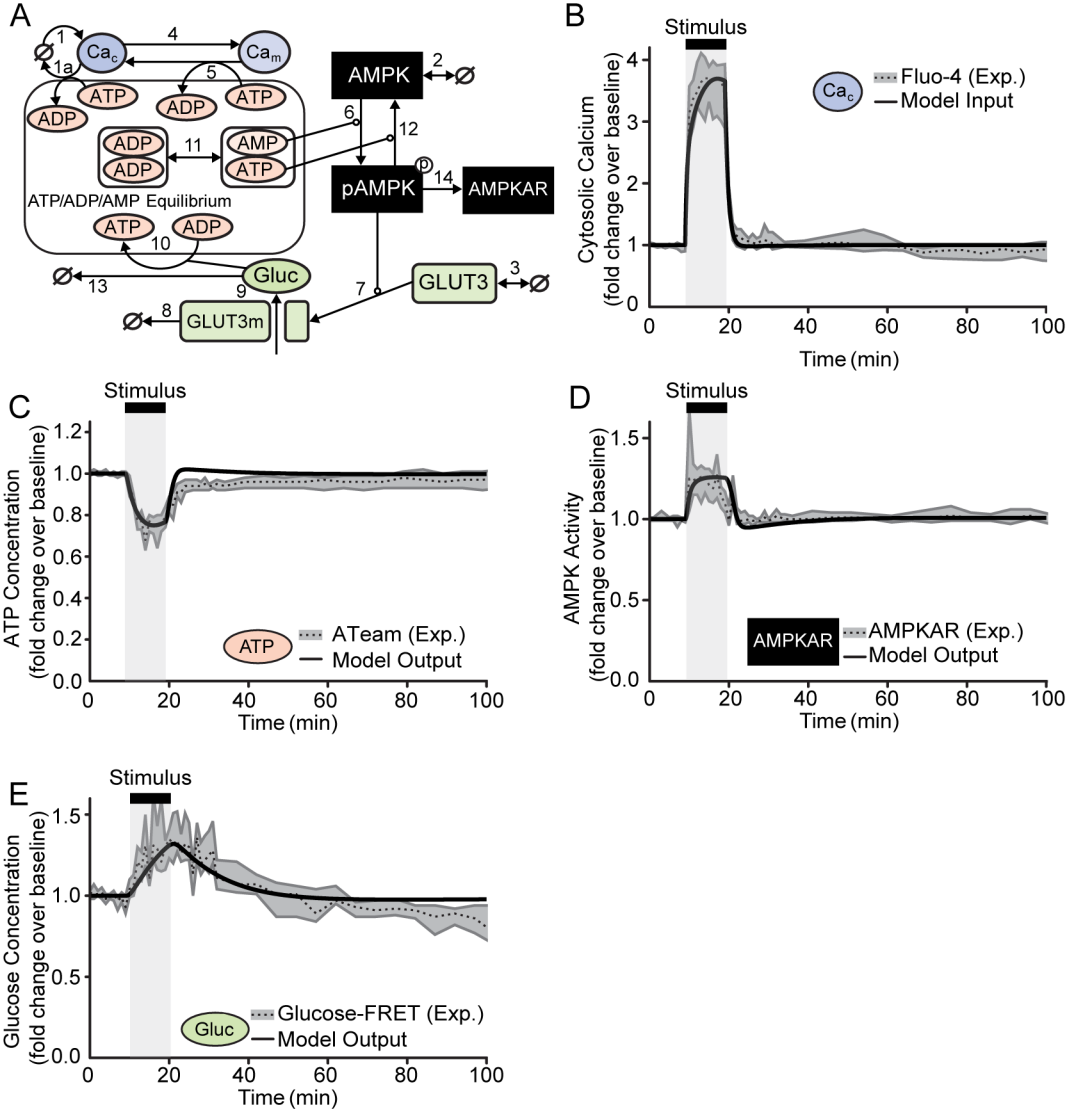
The computational model correctly predicted ATP, AMPK activity and glucose dynamics measured in neurons exposed to glutamate. (A) Model schematic. State variables are described in Table 1, and reaction numbers correspond to those listed in Table 2 and S1 Table. (B-E) Model input and simulations (solid black lines) overlaid on the median and inter-quartile regions (dotted black line, grey shaded area) of previously published fluorescence measurements in single cerebellar granule neurons exposed to glutamate for 10 min [15]. The time of stimulus (model input or glutamate exposure) is marked with a light grey bar. Values were normalised to baseline. (B) A transient (10 min) increase in cytosolic calcium was applied as model input (see Methods and d[Cac]/dt equation in S1 Table), and fitted to fluorescence measurements of cytosolic calcium (Fluo-4 AM) in CGNs exposed to glutamate. (C) The simulated ATP dynamics closely aligned with experimental measurements of intracellular ATP concentration [ATeam is a fluorescent reporter of intracellular ATP concentration; [16]]. (D) The simulated transient activation of AMPK resembled experimental measurements of AMPK activity [AMPKAR is a fluorescent reporter of AMPK activity [17]]. (E) The model also correctly predicted a prolonged elevation of intracellular glucose and its delayed recovery [Glucose-FRET is a fluorescent reporter of intracellular glucose concentration [18]].

We previously demonstrated that AMPK, activated during excitotoxicity-induced energetic stress in primary neurons, increased the surface expression of glucose transporters [12]. We hypothesised that this process would mediate increased glucose uptake, provide substrate to restore depleted ATP, and facilitate neuronal survival. To test this proposed cytoprotective role of AMPK *in silico*, we devised a computational model (Fig 1A). We strived for simplicity by incorporating only the critical processes involved in the response. We reasoned that acute excitotoxicity-induced alterations are severe and rapid, overwriting other physiological or pathological processes that may also be present, and thus constrained our model to a minimal set of equations (Tables 1–2 and S1 Table). We therefore modelled a coarse-grain system of biochemical reactions between ions, metabolites and signalling enzymes incorporating calcium (Ca^2+^) shuttling, ATP/ADP/AMP homeostasis and the afore-mentioned AMPK-mediated increase in glucose import (see Methods for specific implementation details). This reductionist approach and the resultant manageable number of equations enabled steady-state stability analysis to reduce the number of unknown parameters and constrain the free parameter space to represent physiological steady-state in the absence of stress (Table 3 and Methods).

**Table 1:** Steady-state concentrations of modelled state variables.

State Variable	Description	Steady-State Conc. (nM)	Reference
**Ca**_**c**_	Cytosolic Calcium	180	[19]
**Ca**_**m**_	Mitochondrial Calcium	320	[20]
**AMPK**	AMP Kinase	144	[21]
**pAMPK**	Phosphorylated AMPK	81	
**AMPKAR**	AMPK-activity reporter	81	
**GLUT3**	Glucose Transporter 3 (cytosolic)	400	[22]
**GLUT3m**	Membranous GLUT3	233	[22]
**Glucose**	Intracellular Glucose (= ‘Gluc’ / 25)	4.5 mM	[23]
**ATP**	Adenosine Triphosphate	2.7 mM	[24]
**ADP**	Adenosine Diphosphate	200 μM	ATP:ADP ~100:10 [25]
**AMP**	Adenosine Monophosphate	24 μM	ATP:AMP ~ 100:1 [25]

State variables described in the model, their biological description and their steady-state concentrations, which were maintained close to literature values where available (last column), or estimated within physiologically reasonable limits. ‘Gluc’ represents 1/25 of the total glucose concentration (see Methods).

**Table 2:** Reaction (Rx) equations and kinetic flux values for the modelled reaction network.

Rx #	Reaction Equation	Half-Life [min]	k_on_ [s^-1^], [nM s^-1^] or [nM^-1^ s^-1^]	k_off_ **[s**^-1^**] or [nM**^-1^ **s**^-1^**]**	**Reaction Description**
1*	→ Ca_c_	-	19.4	-	Cytosolic calcium influx
**1a**	Ca_c_+ATP→ADP	-	40 x 10^−9^	-	ATP-dependent cytosolic calcium efflux
2*^†^	→ AMPK →	360 [26]	4.6 x 10^−3^	32 x 10^−6^	AMPK turnover
**3**^†^	→ GLUT3 →	900 [27]	125 x 10^−3^	13 x 10^−6^	Turnover of cytosolic glucose transporter 3
4*	Ca_c_ → Ca_m_	-	42	-	Passive diffusion of Ca^2+^ from cytoplasm to mitochondria
5*	Ca_m_ + 0.8*ATP → Ca_c_ + 0.8*ADP	-	169 x 10^−6^	-	Active (ATP-consuming) Ca^2+^ efflux from the mitochondria
6*	AMP + AMPK → AMP + pAMPK	-	1.3	-	AMP-mediated AMPK phosphorylation
7*	pAMPK + GLUT3 → GLUT3m + pAMPK	-	3.7 x 10^−6^	-	pAMPK-mediated translocation of GLUT3 from the cytoplasm to the plasma membrane
8*	GLUT3m →	23	-	0.5 x 10^−3^	GLUT3m degradation
9*	GLUT3m → GLUT3m + Gluc*25	-	4.5 x 10^3^	-	GLUT3m-mediated glucose import
**10**	Gluc + ADP → ATP	-	0.27 x 10^−9^	-	Glucose-mediated ATP production
11*	ADP + ADP < = > ATP + AMP	-	73 x 10^−9^	45 x 10^−9^	Reversible adenylate kinase reaction
**12**	ATP + pAMPK → AMPK + ATP	-	20 x 10^−3^	-	Dephosphorylation of pAMPK
**13**^†^	Gluc →	1.2/25 [28]	-	0.23	Non-ATP-producing glucose consumption
**14**	AMPKAR = k * d(pAMPK)/dt	-	0.17	-	AMPKAR activity factor

Biological reactions, half-life times and kinetic constants of the model. Reaction rates were assumed by using mass action kinetics (Rx 1a, 4–12), first order degradation (Rx 1–3, 8, 13), protein synthesis (Rx 2, 3) and a product term (Rx 14). Values were either maintained close to literature values (references in square brackets), determined from steady-state constraints (marked with *), or tuned during model calibration as detailed in the Methods. The stoichiometric factors in Rx5 and Rx9 are detailed in the model description (Methods).

* k_on_ values determined from steady-state constraints (Table 3)

^†^ k_off_ values determined from half-life (k_off_ = ln(2) / t_1/2_).

**Table 3:** Parameter constraints determined from steady-state (ss) analysis.

**Parameter**	**Steady-State Constraint**
**k**_**on**_**1**	*Ca*_*css*_ *ATP*_*ss*_ *k*_*on*_*1a*
**k**_**on**_**2**	*AMPK*_*ss*_ *k*_*off*_*2*
**k**_**on**_**4**	$\frac{1.\mathrm{25}(ADP_{ss}.Gluc_{ss}.k_{on}\mathrm{10}-ATP_{ss}.Ca_{css}.k_{on}1a)}{Ca_{css}}$
**k**_**on**_**5**	$\frac{1.\mathrm{25}(ADP_{ss}.Gluc_{ss}.k_{on}\mathrm{10}-ATP_{ss}.Ca_{css}.k_{on}1a)}{ATP_{ss}^{0.8}.Ca_{mss}}$
**k**_**on**_**6**	$\frac{ATP_{ss}.pAMPK_{ss}.k_{on}\mathrm{12}}{AMP_{ss}.AMPK_{ss}}$
**k**_**on**_**7**	$\frac{-Glut3_{ss}.k_{off}3+k_{on}3}{Glut3_{ss}.pAMPK}$
**k**_**off**_**8**	$\frac{-Glut3_{ss}.k_{off}3+k_{on}3}{Glut3m_{ss}}$
**k**_**on**_**9**	$\frac{ADP.Gluc.k_{on}\mathrm{10}+Gluc_{ss}.k_{off}\mathrm{13}}{\mathrm{25}.Glut3m_{ss}}$
**k**_**on**_**11**	$\frac{AMP_{ss}.ATP_{ss}.k_{off}\mathrm{11}}{ADP_{ss}^{2}}$

Steady-state analysis determined a number of kinetic parameter constraints, reducing the number of unconstrained free parameters. We imposed these constraints during model analysis to simultaneously represent both the physiological steady-state and the transient perturbation dynamics. The factor 1.25 in k_on_4 and k_on_5 is the reciprocal of the 0.8 stoichiometry in Rx5 (Table 2).

To represent excitotoxic stress, we assumed a transient glutamate stimulus as model input. We mimicked this by a 10 min increase in cytosolic Ca^2+^ (Ca_c_, see Fig 1B, Methods and d[Cac]/dt equation in S1 Table) as previously measured by us and others in cortical and cerebellar neurons in response to NMDA/glutamate [15, 29–31]. As output, the model predicted a rapid increase in mitochondrial calcium (Ca_m_), as measured *in vitro* [29–31]. These elevated Ca^2+^ levels depleted ATP and increased ADP and AMP, resembling excitotoxicity-induced energetic stress [9, 12, 13, 32]. The model further predicted an increase in AMPK activity, consistent with its activation during energetic stress [12, 33]. Finally, the model also predicted an increase in intracellular glucose, in agreement with measurements in primary cultured neurons [15]. This response was explained by increased surface expression of the GLUT3 transporters [12] and led to elevated ATP production and restored energetic homeostasis, representing tolerance to transient excitotoxic stress.

As the modelled dynamics qualitatively agreed with experimental observations, we next assessed whether the model could quantitatively resemble the experimental kinetics of neurons exposed to transient excitotoxicity. We remodelled single-cell measurements, assuming small variations of model parameters to represent cell-to-cell variability, rather than remodelling biochemistry experiments, as critical cellular dynamics can often be overlooked in population-level measurements [34, 35]. Indeed, calibrated model results revealed that the maximum ATP depletion and its rapid recovery closely resembled single cell data (Fig 1C), suggesting that ATP depletion during excitotoxicity is primarily affected by ionic imbalance, and that an AMPK-mediated increase in intracellular glucose may be sufficient to rapidly recover ATP levels. The simulated kinetics of AMPK activity also aligned with single-cell measurements (Fig 1D), although the model could not sufficiently explain the recovery of AMPK activity prior to stimulus termination as observed in some cells. Finally, the simulated glucose recovery was markedly slower than the recovery of ATP and AMPK activity (25.1 min vs. 2.4 and 2.9 min respectively) and accurately resembled the extent of the delayed recovery measured in single neurons (Fig 1E). This suggested that the intracellular glucose concentration during and following transient excitotoxicity may be primarily regulated by the AMPK-mediated ATP production incorporated in our model design.

### Multiple model simulations closely resemble experimental cell-to-cell heterogeneity

**Fig 2:**
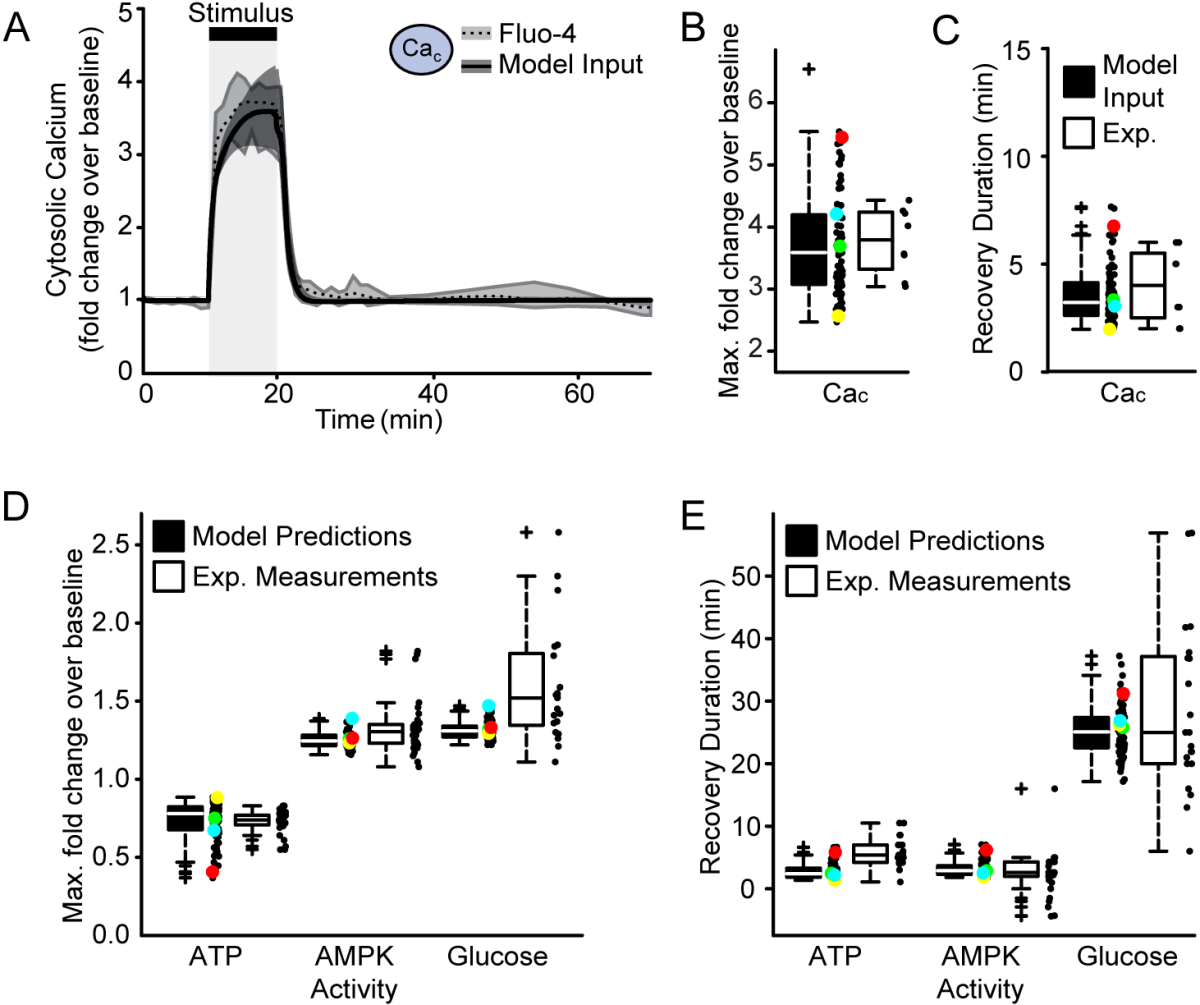
Multiple model simulations with varied parameter sets represented the experimental cell-to-cell heterogeneity. (A-E) Cell-to-cell heterogeneity was modelled by varying parameter values and input characteristics (magnitude and duration of the applied calcium influx) and performing multiple simulations. (A) The median and inter-quartile regions of all model inputs (solid black line within dark grey region) well resembled the median and inter-quartile regions (black dotted line within light grey region) of previously published fluorescent measurements of cytosolic calcium [Ca_c_; [15]]. (B-E) For each simulation, the metrics shown in (B-E) were calculated. The coloured data points link these simulations across the figures. The green data points were predicted with the parameter set as listed in Tables 1 and 2. The parameter sets predicting the yellow, cyan and red data points are listed in S2 Table. (B,C) The predicted variability in the (B) maximum fold change and (C) recovery duration of the model input (black box) closely matched experimental measurements (white box). Recovery duration was calculated as the time taken for the signal to recover to ±2% of baseline signal. (D) Box- and scatter-plots of the minimum ATP, maximum AMPK activity and maximum glucose fold changes during the excitotoxic stimulus, calculated from multiple model predictions and experimental measurements (Exp.) from [15]. (E) Box- and scatter-plots of the post-excitotoxicity recovery duration of the ATP, AMPK activity and glucose levels as calculated from model predictions and experimental measurements (Exp.) from [15].

We next aimed to model cell-to-cell heterogeneity in our system. We therefore performed multiple simulations and varied independent parameters within ±20% of their original values (Tables 1 and 2). We also varied the magnitude and duration of the excitotoxic stimulus to represent asynchronous responses or intrinsic variations in ion channel or pump expression. The resultant variability in the model input closely matched the experimental Ca_c_ profile (Fig 2A). Specifically, the distribution of the Ca_c_ maximum fold change, and the time taken for the signal to return to baseline (‘recovery duration’), accurately represented the experimental variability (Fig 2B and 2C). Under these conditions, the predicted distribution of the ATP, AMPK activity and glucose fold-changes also well represented single-cell measurements, with some discrepancy in the glucose response (Fig 2D). Likewise, the model accurately predicted the experimental variability in the recovery of AMPK activity (Fig 2E). Although the model resembled the primarily rapid recovery kinetics of ATP, it could not explain the slower kinetics as the signal approached baseline (Fig 1C). Strikingly, the model also predicted the delayed recovery of glucose (Fig 2E). However, while well resembling the experimental heterogeneity within the inter-quartile regions (box-edges), the model was not able to describe the spurious events governing the dynamics of the outlying cells. Nevertheless, we conclude that our computational model can closely predict the cell-to-cell heterogeneity within a neuronal population during transient excitotoxicity.

### Sensitivity analysis suggests that the dynamics of glucose import critically regulate the rate of glucose recovery following transient excitotoxicity

**Fig 3:**
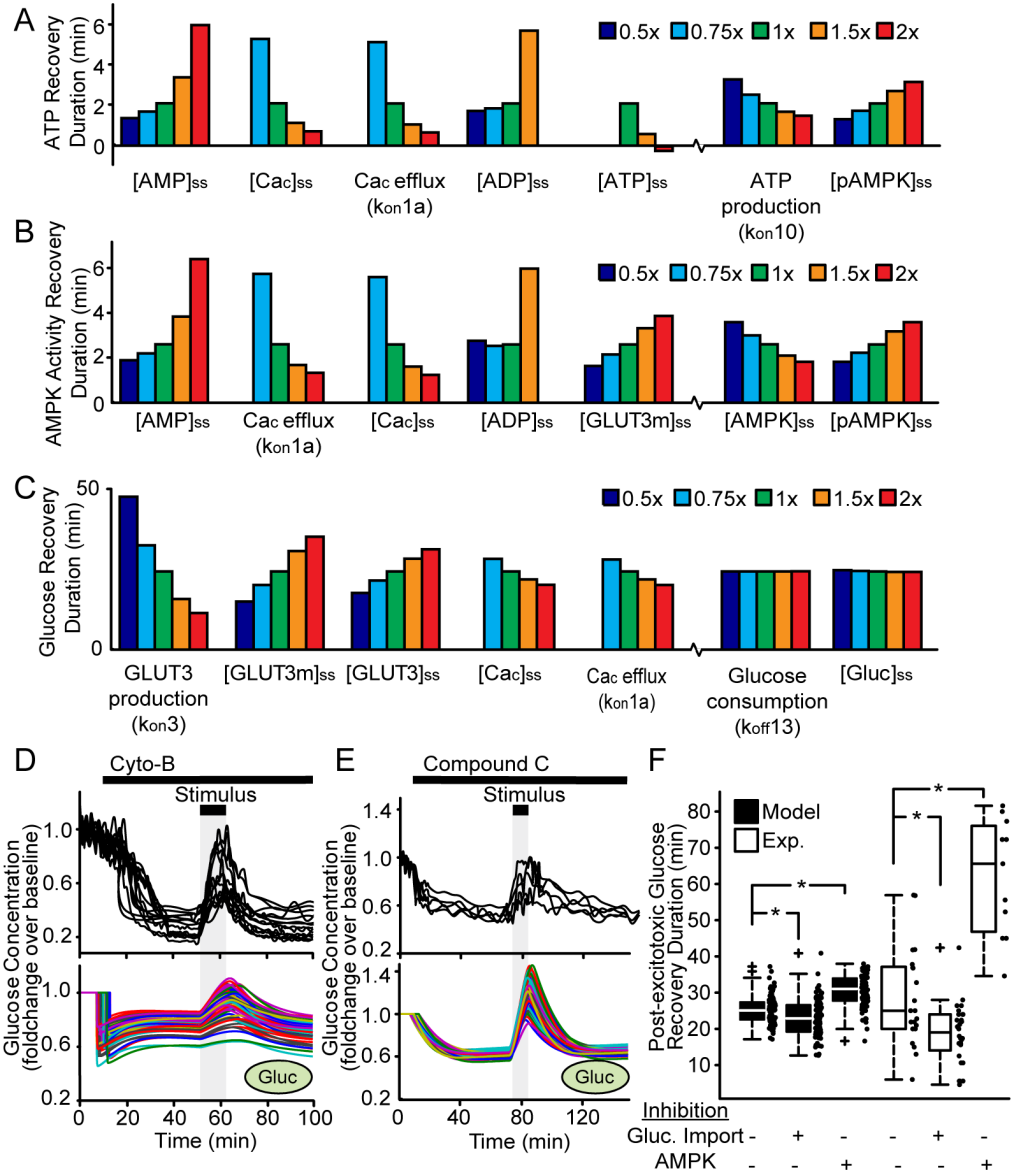
Sensitivity analysis indicated that glucose import dynamics are critical to the post-excitotoxic glucose recovery. (A-C) Parameters were varied by 0.5 (navy), 0.75 (light blue), 1 (green), 1.5 (orange) and 2 (red) times the steady-state values listed in Tables 1 and 2, and the effect was calculated for the post-excitotoxic recovery duration of the (A) ATP, (B) AMPK activity and (C) Glucose signals. The varied parameter is written under each bar chart. The 5 parameters with the greatest effect on each metric are shown (left to right in order of effect), along with other parameters mentioned in the text. Data were omitted for parameter values at which the modelled state variables did not return to baseline within the simulation time (100 min). (D, E) Experimental traces (top panels) and multiple model simulations (bottom panels) of intracellular glucose concentration with either (D) glucose import or (E) AMPK inhibited prior to exposure to a transient excitotoxic stimulus. (D) Glucose import was inhibited by exposure to Cytochalasin B or by reduction of the modelled glucose import kinetics (Rx 9). (E) AMPK activity was inhibited by exposure to Compound C or by reduction of the modelled AMPK phosphorylation kinetics (Rx 6). Compound C experiments have been published previously [Fig 6A from [15]]. (F) Box- and scatter-plots of the glucose recovery duration with and without glucose import or AMPK inhibition (* ranksum p < 0.05).

To identify parameter changes that most impact the system response, we performed a sensitivity analysis. We varied single parameter values to 0.5, 0.75, 1, 1.5 and 2 times their values listed in Tables 1 and 2, under the constraints of steady-state conditions (see Methods). We found that the predicted duration of ATP recovery was primarily affected by the steady-state concentrations of adenine nucleotides ([AMP]_ss_, [ADP]_ss_ and [ATP]_ss;_ Fig 3A), while high [ATP]_ss_ predicted ATP recovery prior to stimulus termination, an effect seen in some single-cell experiments [15]. Interestingly, low [ATP]_ss_, high [ADP]_ss_ and low [Ca_c_]_ss_ predicted that ATP would not return to baseline after the stimulus (missing bars). Similar parameters also more critically regulated AMPK recovery compared to AMPK-related parameters (Fig 3B), indicating that ATP concentration and AMPK activity are tightly coupled. This may be expected, as the ATP:AMP ratio strongly regulates AMPK phosphorylation and activity. Interestingly, variations in GLUT3-related parameters were predicted to severely impact the rate of glucose recovery (Fig 3C), suggesting that the dynamics of glucose import were more critical than the dynamics of glucose consumption (k_on_10, k_off_13).

We therefore modelled the effect of GLUT3 inhibition on glucose dynamics by reducing the respective import kinetics (reaction Rx 9; k_on_9). As a result, the model predicted a decrease in intracellular glucose levels, although to a lesser extent than in *de novo* measurements of CGNs exposed to Cytochalasin B, an inhibitor of glucose transport (Fig 3D). The model also predicted a trend towards a more rapid recovery of post-excitotoxic glucose levels, and this prediction was validated with additional experimental data not used in model calibration (Fig 3F). We further investigated the effect of AMPK inhibition by reducing the rate of AMPK phosphorylation (Rx 6; k_on_6), and found that the model predicted a prolonged recovery of glucose to baseline. These predictions were qualitatively validated by measurements in cerebellar neurons treated with the AMPK inhibitor Compound C (Fig 3EF here and Fig 6a from [15]). The model did not predict the full effect of these inhibitory effects on glucose dynamics, likely due to off-target pharmacological effects on processes excluded from model design.

### The computational model predicted the necrotic bioenergetic collapse induced by severe excitotoxic stress

**Fig 4:**
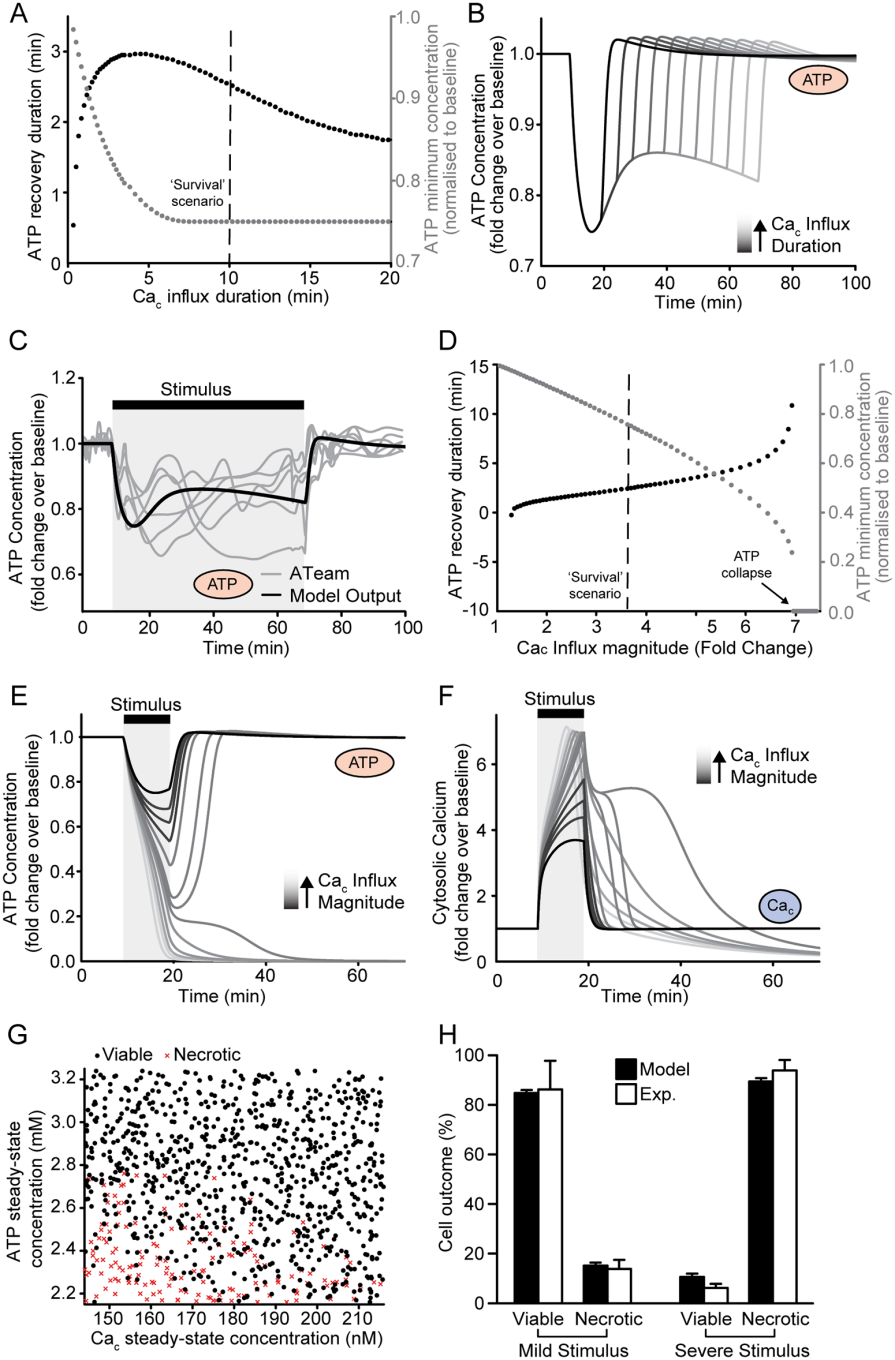
The computational model can represent the bioenergetic collapse induced by severe excitotoxicity, modelling neuronal necrosis. We investigated the predicted responses following more severe excitotoxic stimuli by increasing the (A-C) duration or (D-F) magnitude of the applied calcium influx (model input). (A) Prolonging the Ca_c_ influx beyond 5 min did not predict further depletion of ATP beyond a minimal level (grey dots). In contrast, longer periods of Ca_c_ influx were predicted to speed up ATP recovery (black dots). The calcium influx for which the graphs in Fig 1 were plotted is indicated with a black dashed line. (B) ATP was predicted to initiate recovery during longer periods of Ca_c_ influx. (C) The predicted ATP dynamics (black line) during a 60 min excitotoxic stimulus were validated with *de novo* fluorescent measurements of ATP concentration (ATeam) in CGNs exposed to glutamate for 60 min (grey lines) (D) More severe calcium influx was predicted to exacerbate ATP depletion (grey dots) and prolong its recovery (black dots). Behaviour consistent with neuronal necrosis was predicted for maximal values of calcium influx > 7 fold change, where the ATP concentration collapsed to ~0, and did not recover. (E) The model predicted characteristic switch-like behaviour in the ATP dynamics leading to necrotic energetic collapse on severe calcium influx. (F) At magnitudes of calcium influx that induced ATP collapse (lighter grey lines), the model also predicted altered calcium dynamics. (G) The steady-state concentrations of ATP and Ca_c_ well predicted the simulation outcome (viable/necrosis) following calcium influx. (H) The percentage of neurons predicted to undergo necrosis or to remain viable following an excitotoxic stimulus were similar to levels measured in populations of cortical neurons exposed to NMDA. Experimental data from [37] Fig 1F, and the bar charts display mean ± SEM.

We next investigated the predicted response to more severe excitotoxicity. We first investigated the relation between Ca_c_ influx duration and maximal ATP depletion, yet no correlation was observed (Fig 4A, grey scatter points), consistent with literature data [32]. However, ATP levels were predicted to partially recover during longer stimuli (Fig 4B). To validate this, we performed *de novo* single-cell experiments of ATP during a 60 min excitotoxic exposure which confirmed the predicted dynamics (Fig 4C). We next increased the magnitude of Ca_c_ influx and found that the model predicted exacerbated ATP depletion and delayed ATP recovery (Fig 4D; grey and black scatter points respectively). Strikingly, ATP collapse, considered to represent neuronal necrosis [9, 36], was predicted at a maximum Ca_c_ influx of around 1.2 μM, with a minimal ATP relative to baseline of around 0.3. We noted that this fold change was similar to experimental measurements of the ATP fluorescent signal (ATeam) during cell death in CGNs [15]. Interestingly, simulations predicting ATP collapse (Fig 4E) also predicted a significantly altered Ca_c_ response (Fig 4F).

We further investigated the parameter sets predicting this characteristic switch-like behaviour. Similar to the above procedure, we performed multiple simulations by varying the parameter sets to represent heterogeneous intracellular conditions, and assumed neuronal necrosis upon ATP depletion below 35% of baseline. We found that the steady-state concentrations of ATP and Ca_c_, along with kinetic constants constrained by these concentrations, were the primary parameters influencing the transition to necrosis upon subsequent calcium influx. When plotted against each other, these two parameters well predicted simulation outcome (Fig 4G). Based on these findings, we finally explored whether the model could also predict the levels of excitotoxic necrosis in neuronal populations (Fig 4H). The model predicted a similarly low level of necrotic cell death as measured in populations of cortical neurons exposed to 100 μM NMDA for 5 min (‘Mild stimulus’; 15 ± 1.3% vs. 14 ± 4%) [37]. A more severe stimulus (ca_mag = 60 or 300 μM NMDA for 60 min), was predicted to increase necrosis to similar levels as measured in the cortical populations (89 ± 1.3% vs. 94 ± 4%). Taken together, these data suggested that the model may successfully represent neuronal necrosis following severe and prolonged excitotoxicity.

## Discussion

In this study we established and analysed a computational model of the AMPK-mediated bioenergetic and neuroprotective response to transient and prolonged excitotoxic stress. The model recapitulated the acute energetic response of primary neurons exposed to glutamate, a heavily investigated experimental model [6, 9, 12, 15, 38, 39]. Despite minimising the number of variables and reactions during model development, the model surprisingly well resembled bioenergetic responses at both the single-cell and population level, allowing us to surmise that the modelled reactions describe the critical regulatory processes underlying the observed behaviour.

Modelling transient and mild cytosolic Ca^2+^ influx accurately predicted the rapid recovery and low cell-to-cell heterogeneity of ATP levels and AMPK activity, suggesting that a neuroprotective AMPK-mediated glucose increase could play a key role in ATP recovery. We cannot discount, however, that other factors (such as kinases with similar kinetics to AMPK) may also contribute. Notably, prolonged cytosolic Ca^2+^ influx did not predict exacerbated ATP depletion. Rather, predictions that ATP levels would plateau and even begin to recover during such stimuli were validated experimentally. In such instances, mitochondrial Ca^2+^ sequestration may prevent further ATP depletion by stabilising cytosolic Ca^2+^ levels, or facilitate ATP replenishment by elevating mitochondrial respiration through Ca^2+^-dependent dehydrogenases [6, 40]. In contrast, severe ATP depletion was predicted upon higher cytosolic Ca^2+^ influx, representing the bioenergetic collapse observed during excitotoxic necrosis [9, 36]. Here, the altered cytosolic Ca^2+^ dynamics predicted by our model resembled calcium deregulation, a process associated with the loss of Ca^2+^ homeostasis, severe mitochondrial membrane depolarisation and failure of mitochondrial bioenergetics seen *in vitro* [8, 11, 30, 41]. Interestingly, the steady-state concentrations of ATP and cytosolic Ca^2+^ significantly influenced this transition to bioenergetic collapse following subsequent Ca^2+^ influx. We note that varying the steady-state concentration of ATP also alters the energy charge of the cell. To validate this result, future experiments could investigate correlations between intracellular ATP or Ca^2+^ concentrations and subsequent necrotic cell death following glutamate exposure. Finally, the model closely predicted the amount of necrosis measured in neuronal populations at different insult severities, indicating that acute excitotoxic necrosis is mediated primarily by cytosolic calcium influx [42].

In striking contrast to the rapid dynamics of ATP levels and AMPK activity, the model also correctly predicted the prolonged elevation and delayed recovery of intracellular glucose following transient excitotoxic stress. Model analysis suggested that these dynamics were critically regulated by the efficacy of GLUT3 transporters. Indeed, neurons retain their capacity for increased glucose import following transient excitotoxic stress [6], likely mediated by AMPK [12]. Excess glucose availability may provide a mechanism whereby cells prioritise the maintenance of resources for ATP recovery, even if ATP is only required to ensure apoptotic, rather than necrotic, cell death [36]. However, as prolonged hyperglycaemia may induce lactic acidosis and production of toxic reactive oxygen species [43], mechanisms are required to optimise glucose processing. Indeed, we recently found that neurons capable of more quickly restoring baseline glucose levels may survive longer following excitotoxicity (immediately necrotic neurons were excluded from this analysis) [15]. This indicates that tight regulation of any altered glucose metabolism may be crucial to neuronal survival downstream of the acute response. To this end, AMPK activates PFK2 to stimulate glycolysis in cardiac ischemia [44], and likely functions similarly in neurons following excitotoxicity [45, 46]. The role of AMPK in modulating intracellular glucose was further emphasised as our compact model was able to sufficiently predict the higher experimental cell-to-cell variability of the glucose response, and qualitatively predicted the impact of AMPK inhibition on glucose recovery dynamics. Indeed, incorporating glucose regulation processes independent from AMPK did not improve model performance (S1 Fig and S3 Table), also indicating that additional complexity may not enhance model utility. This does not rule out the possibility of other mechanisms of glucose uptake, mobilisation or processing during neuronal excitotoxicity, such as recently proposed intracellular glucose stores [15, 47], but suggests that these mechanisms may be AMPK-dependent. AMPK may therefore address the elevated energy demand following excitotoxicity by both enhancing substrate supply from multiple sources, and by regulating the metabolism of this increased supply, with any imbalance leading to toxic side-effects. The model described herein can now be utilised as an additional tool to investigate such mechanisms, complementary to existing detailed models of specific neuronal bioenergetics components [48, 49].

One of the key goals of this study was to model the complex system of neuronal energetics during excitotoxicity by a reduced but essential set of differential equations. Such a reductionist approach enabled us to reduce the number of unconstrained parameters and allowed us to capture the primary characteristics of energetic perturbation during the specific and timely well-confined process of transient excitotoxicity. Our model is also relatively straight-forward to implement and fast to run, making it amenable to further dissemination. We are aware of the limitations of such an approach, however, and cannot discount that some of the biological reactions assumed as present and constant in our model may contribute to the observed behaviour. Nevertheless, the ability of the compact set of equations to reproduce the observed experimental behaviour suggests that these equations, and the reactions they describe, may critically regulate the acute energetic response in the particular environment of excitotoxic stress.

In conclusion, we have developed a reduced computational model that closely resembles the acute bioenergetic responses to transient excitotoxicity, indicating a key role for AMPK and suggesting that these responses are critically regulated by a minimal set of core reactions, leading to a robust bioenergetic response to transient excitotoxic stress.

## Materials and Methods

### Computational modelling of molecular pathways

Excitotoxicity and the neuroprotective signalling of post-glutamatergic AMPK activation was modelled by a system of biochemical reactions based on mass-action kinetics. Reactions, initial concentration and kinetic parameters are given in Fig 1A and Tables 1–2 and S1 Table and reactions were assembled as follows. First, Ca^2+^ homeostasis was assumed to be maintained in the absence of excitotoxic stress by shuttling between the extracellular space and the cytosol (Rx 1). The extrusion of Ca^2+^ from the cytosol was modelled to consume ATP, representing ATP-dependent ion pumps such as the plasma membrane Ca^2+^ ATPase (PMCA, Rx 1a). Second, transcription and proteasomal/lysosomal degradation were assumed to maintain constant cytosolic levels of unphosphorylated AMPK (Rx 2) and those of the most highly expressed neuronal glucose transporters, GLUT3 (Rx 3) [50]. These reactions were not modelled to consume ATP, as such ATP consumption likely contributes little to the pathological energetic stress mediated by excessive intracellular calcium accumulation, which has been shown to be the primary mediator of excitotoxic damage [4, 42]. Moreover, these processes (transcription, translation, degradation) operate over longer time-scales than that simulated here. Specifically, the half-lives of AMPK and GLUT3 production/degradation processes were set to literature values of 360 and 900 min, while the duration of transient excitotoxic injury was measured and modelled with time frames < ~100 min. In agreement with this, previous studies from our laboratory in primary neurons exposed to transient excitotoxic stress measured no significant changes in AMPK or GLUT3 protein concentrations [12], or in proteasome-mediated degradation [51].

Next, mitochondrial Ca^2+^ import (Rx 4) was modelled as a passive diffusion process [8], while Ca_m_ extrusion into the cytosol was assumed to require ATP by transporting ions against the electrochemical gradient of the inner mitochondrial membrane (Rx 5) [8]. The stoichiometry of Rx 5 (0.8 molar units of ATP consumed to ADP for each Ca^2+^ exported from the mitochondria) was calculated by assuming a 3:1 stoichiometry for Na^+^/Ca^2+^ exchangers [52], a 1:1 stoichiometry for H^+^/Na^+^ exchange, and a ratio of 3.67 for the proton-motive (H^+^) force to produce one ATP molecule by the ATP synthase [53, 54]. AMPK phosphorylation status was mediated by AMP and ATP (Rx 6, 12) [25] with basal phosphorylated AMPK (pAMPK) activity resulting from constitutively present AMP, as observed [12, 55]. Ratios between ATP, ADP and AMP levels were further maintained by the adenylate kinase reaction (Rx 11) [25, 56].

To incorporate the AMPK-mediated increase in GLUT3 surface expression as previously demonstrated by us [12], pAMPK was modelled to mediate the translocation of cytosolic GLUT3 to the plasma membrane (GLUT3m; Rx 7). GLUT3m was constitutively present and underwent proteasomal degradation (Rx 8). GLUT3m facilitated glucose import (Rx 9) from an unlimited extracellular pool. This uni-directional transport rendered the proportional shift in the cytosolic glucose balance upon excitotoxic stress. Non-ATP-producing glucose consumption, such as in the pentose phosphate pathway, was modelled as a first order consumption process (Rx 13). ‘Gluc’ represented 1/25 of the total glucose concentration, combining glycolytic and mitochondrial ATP production where consumption of one glucose molecule converted 25 ADP to 25 ATP (Rx 10). This implementation choice allowed us to avoid otherwise high exponential factors (25 ATP translates to [ATP]^25^ when mass action is applied), circumventing numerical issues in ODE solving. The value of 25 reflects the fact that some glucose consumed in ATP-producing reactions (i.e. glycolysis) will be shunted to lactate (producing only 2 ATP molecules compared to 36 when glucose is oxidised in the mitochondria). This was calculated from data in [15], where 70% of ATP during acute glutamate exposure derived from mitochondrial oxidative phosphorylation (based on additional glutamate-induced ATP depletion in the presence of oligomycin). This value is similar to values found in the literature [57, 58]. We did not implement an effect of mitochondrial Ca^2+^ on ATP production, as the precise effects of excessive mitochondrial Ca^2+^ sequestration are not clear [7, 59–61], and any potential increase in TCA cycle activity is likely insufficient to address the severely increased excitotoxic ATP demand [38, 60]. Finally, pAMPK was modelled to activate AMPKAR, the fluorescent reporter of AMPK activity (Rx 14) [17]. Inhibition of glucose import by Cytochalasin B was modelled by reducing glucose import kinetics (Rx 9, k_on_9 reduced to 60% of its steady-state value), and inhibition of AMPK activity by Compound C was modelled by reducing AMPK phosphorylation kinetics (Rx 6, k_on_6 reduced to 10% of its steady-state value). Reactions were translated into a set of ordinary differential equations (ODEs) and solved in MATLAB R2007B (The Mathworks, UK), using the in-built adaptive step Runge-Kutta solver ode15s [62]. Model codes are available from the authors on request.

### Steady-state concentrations and kinetic parameters

Steady-state concentrations and kinetic parameters were maintained close to literature values where available (Tables 1 and 2, 12 of 29 parameters). Values measured in neurons/brain were preferred over other data where possible. Resting Ca_c_ and Ca_m_ values of 180 nM and 320 nM, respectively, resemble measurements in hippocampal neurons and rat brain capillary endothelial cells [19, 20]. The steady-state AMPK concentration (144 nM) was taken from rat liver [21]. Intracellular glucose (4.5 mM) was consistent with neurons measured under the same external glucose media as our CGN cultures (15 mM) [23]. We note that 4.5 mM is greater than the K_m_ of Hexokinase (HK) for glucose [23, 28], suggesting that changes in the intracellular glucose concentration may not be translated into increased glucose utilisation. However, the kinetics of HK and other glycolytic enzymes such as phosphofructokinase are also sensitive to ATP concentration, and the functional maximal activity of HK may change in relation to glucose import kinetics [28] or according to the location or ATP source of the relevant HK isoform [63]. In the detailed model of glucose transport by Simpson *et al*, the authors related glycolysis and glucose transport through a coupling factor and acknowledged that experimentally measured high glucose levels would not be possible otherwise [23]. We modelled ATP-producing glucose utilisation by non-saturable mass action kinetics. ATP (2.7 mM) has been measured in rat neurons [24] and the ATP:ADP:AMP ratio was maintained ~100:10:1 as observed [25], with the resting adenylate charge ~0.95 as expected in the brain [64]. GLUT3 and GLUT3m steady-state concentrations approximated measurements in rat cerebellar neurons [22]. The ratio of [GLUT3]_ss_:[GLUT3m]_ss_ was assumed to be ~0.71:1.13 at steady-state, as measured in hippocampal neurons at rest [65]. As expected, this ratio changed upon stimulus onset as calculated by our model. AMPK and GLUT3 degradation half-life times were based on measurements in COS-7 cells and myotubes [26, 27], respectively. The glucose half-life was measured in rat cerebella [28]. A further 9 parameters were determined by steady-state constraints (Table 3 & ‘Steady-State and Stability Analysis’ below), leaving 8 parameters that were tuned to single-cell experimental data (see ‘Parameter Estimation’ below). Of note, kinetic constants following calibration closely aligned with other literature data. Specifically, kinetic constants for AMPK phosphorylation and dephosphorylation (Rx 6, 12) approximated measurements in HeLa cells [66]. The kinetics of glucose import (Rx 9) aligned with measurements in rat cerebellar neurons [23], and the adenylate kinase reaction (Rx 11) remained at equilibrium at steady-state.

### Steady State and Stability Analysis

Under physiological conditions in the absence of stress and noise, state variables were assumed to remain constant over time, resembling the fact that negative feedback from ATP promotes pAMPK dephosphorylation and maintains Ca^2+^ equilibrium. Steady-state calculations from this biological assumption allowed us to reduce the number of unconstrained independent parameters (Table 3). These constraints were maintained throughout parameter tuning and model analysis. Hence, whenever independent parameters were varied, dependent parameters were updated accordingly. In addition, the defined steady state was required to be stable, fulfilling the biological requirement that the physiological state is robust against small perturbations such as induced by subliminal stresses or accidental enzymatic activation [67]. Stability analysis was performed as previously described, calculating the Jacobian associated with the ODE system and requiring that all its eigenvalues have negative real parts [68]. The Jacobian was further reduced to a 9 x 9 matrix by assuming preservation of the total adenosine phosphate pool ATP+ADP+AMP = A_tot_ = 2.924 mM [56]. By this procedure, local stability was guaranteed in the absence of stress, despite the presence of constitutively activated molecules that are observed experimentally [12]. Mathematical analysis was performed in Mathematica (Wolfram Research, Oxfordshire, UK).

### Computational modelling of transient glutamate excitotoxicity

To model transient glutamate excitotoxicity, a temporary (10 min) increase in cytosolic Ca^2+^ (Ca_c_) was assumed by

$${\frac{d[Ca_{c}]}{dt}}_{\left(input\right)}=ca\_mag+\frac{d[Ca_{c}]}{dt},$$

with the parameter ca-mag tuned to fit the simulated Ca_c_ influx profile to experimental single-cell Ca^2+^ measurements in rat cerebellar granule neurons (CGNs) during 10 min glutamate exposure [15]. Specifically, ca-mag was calculated as *k*_*on*_1a*Ca_css_*ATP_ss_*[Ca_Fold_*ATP_*Fold*_-*1]* = 34.5, with Ca_Fold_ and ATP_Fold_ respectively set to 3.7 and 0.75 according to the mean experimental Ca^2+^ and ATP fold changes in Figs 1C and 2C from [15]. Ca-mag was subsequently varied to model different severities of excitotoxic calcium influx as indicated.

### Parameter estimation and model calibration to single-cell fluorescence data

Independent model parameters not constrained by steady-state requirements were tuned to remodel cytosolic Ca^2+^, ATP concentration, AMPK activity and glucose concentration from single-cell fluorescence measurements in primary rat CGNS exposed to 10 min glutamate [15]. To exclude extraneous contributions from dying cells, only experimental traces from neurons that remained viable > 100 min after stimulus were used. Traces were normalised to the average of their first 8 time points. Modelled state variables were normalised to their steady-state concentrations.

To tune the model output to experimental data (Fig 1), independent parameters were varied within narrow ranges around their literature values, where available (listed in Tables 1 and 2). The remaining independent parameters were varied within defined ranges as suggested in [69] and to maintain positive kinetic constants (pAMPK 1–1000 nM; k_on_1a 1x10^-10^–1x10^-5^ nM^-1^ s^-1^; k_on_3 20x10^-3^–140x10^-3^ nM s^-1^; k_on_10 0.1x10^-9^–10x10^-6^ nM^-1^ s^-1^; k_on_12 1x10^-6^–1x10^3^ nM^-1^ s^-1^; k_off_11 0.1x10^-9^–7x10^-6^ nM^-1^ s^-1^). For each parameter set, the 9 dependent kinetic parameters were updated to maintain the system’s steady-state, allowing us to simultaneously fit both the steady-state and transient perturbation dynamics. Parameter sets returning negative kinetic parameters were excluded. For each state variable, a residual (ε) was calculated between the medians of the experimental data and the model prediction -

ε = |Value(experimental)- Value(model)|.

The glucose ε was calculated for the periods outside the time of excitotoxic stimulus, as the glucose-FRET signal may exhibit glucose-independent sensitivity during such stimuli [15]. An objective function was calculated by minimising the sum of all ε over each state variable. The best-fit parameter set is given in (Tables 1 and 2).

### Modelling cell-to-cell heterogeneity and a population response

To simulate cell-to-cell heterogeneity, we assumed the independent kinetic parameters to vary by ± 20% of the basal values in Tables 1 and 2, and updated dependent parameters by requiring a stable steady state as described above. The resulting average coefficient of variability for all parameters was 0.15, consistent with protein expression variability measured over 50–100 h in mammalian cells [70]. The magnitude and duration and onset time of the excitotoxic input was varied by ±10% to model the lack of synchrony to drug exposure or intrinsic expression differences of key molecules such as ion pumps or NMDA receptors. To represent a population tolerant to excitotoxic stress (Fig 2) parameter sets predicting an excessively delayed return to homeostasis (> 80 min) were excluded. As output metrics for each simulation we extracted the maximum or minimum values of ATP, glucose concentration and AMPK activity, and the duration of their recovery to baseline. The predicted recovery duration was calculated as the time taken to return to ±2% of baseline value following stimulus termination. For the population response simulated in Fig 4G and 4H, we performed similar parameter variations and classified simulated cell fate as necrotic if [ATP] decreased below 35% of baseline. Predictions were compared to experimental data from [37] Fig 1F. Neurons defined as ‘DCD’ were considered viable here, to compare neurons undergoing acute necrosis with those surviving the acute phase. Predictions for the ‘mild’ stimulus (ca-mag = 34.5 ±10%, duration = 10 min) were compared to experiments performed with 100 μM NMDA exposure for 5 min. Predictions for the ‘severe’ stimulus (ca-mag = 60 ±10%, duration = 60 min) were compared to 300 μM NMDA exposure for 60 min.

### Parameter Sensitivity Analysis

To study parameters that were most influential on the model output (Fig 3A–3C), each was varied by a factor of 0.5, 0.75, 1, 1.5 and 2 times relative to values from Tables 1 and 2. As above, dependent kinetic constants were updated, output metrics were measured and parameters were ranked according to the maximum difference of each metric value over all simulations. To identify the parameters that most influenced the switch between viable and necrotic cell fate (Fig 4G), an entropy based method was used to analyse parameter sensitivity [71]. This is a probabilistic method discriminating between two outcome variables (viable/necrotic) based on the information content (input parameter set). Similar to the population response above, we performed 1000 simulations and classified cell fate in each simulation as necrotic, if [ATP] decreased below 35% of baseline, or viable otherwise. Parameters responsible for a higher change in entropy when comparing outcomes (viable/necrotic) were considered to have more predictive power. These parameters were identified using information gain criteria (Kullback-Leibler divergence).

### Single-cell fluorescent measurements in rat primary cerebellar granule neurons and ethical approval

Previously published experimental data [15, 37] are clearly referenced in the figure legends and text. *De novo* experiments in Figs 3D, 3F and 4C were performed as previously described [15] under a license granted to Dr. Niamh Connolly by the Irish Department for Health & Children and with ethics approval from the RCSI Research Ethics Committee (REC797). 10 μM Cytochalasin B, purchased from Sigma-Aldrich (Fluka, Ireland), was added after 10 min baseline recording.

### Statistical analysis

Boxplots display lines at the lower, median and upper quartile values, with whiskers extending to values within 1.5 times the inter-quartile range. Scatter points show measurements from single neurons/simulations. Bar charts display mean ± SEM. Differences between the medians was calculated by a rank sum test, with p < 0.05 considered significant. All experiments were performed on neurons from at least 3 independent preparations.

## Supporting Information

S1 Fig Incorporation of additional glucose regulation did not improve model performance. (A) Model extension 1 additionally incorporated enzymatic (phosphofructokinase, PFK) regulation of ATP production (Rx 10) via ATP-mediated negative feedback (Rx 16) but (B) model performance was not improved over the original model, as illustrated by the predicted glucose dynamics (black line), overlaid on single-cell fluorescence measurements from [15]. (C) Model extension 2 implemented a reversible glucose store (Rx 15), as may be provided by the endoplasmic reticulum or via glycogen metabolism, but (D) did not alter model performance.
(TIF)

S1 Table Model flux expressions, differential equations and stoichiometric matrix. Numbered flux expressions align with reactions in Fig 1A and Table 2. The differential equation for cytosolic calcium (Ca_c_) includes the ca_mag term applied as model input. The input was applied between t > ca_onset and t < ca_onset + ca_duration. Ca_onset and ca_duration were set to 10 min, and were varied around this value to simulate fluctuations in cellular response.
(PDF)

S2 Table Parameter sets from Fig 2B–2E. Parameter sets from three simulations as highlighted by the yellow, cyan and red data points in Fig 2B–2E. All metrics displayed in Fig 2B–2E were calculated for each simulation.
(PDF)

S3 Table Model extensions—parameters, equations and steady-state constraints altered from original model. Flux expressions, differential equations, steady-state concentrations and kinetic constants for each model variant are listed here if they differ from the model described in the main paper. See Tables 1–3 and S1 Table to compare with main model.
(PDF)
