# Supplementary figures and images for: Computational Analysis of AMPK-Mediated Neuroprotection Suggests Acute Excitotoxic Bioenergetics and Glucose Dynamics Are Regulated by a Minimal Set of Critical Reactions
Source: PLoS One. 2016 Feb 3;11(2):e0148326. doi: 10.1371/journal.pone.0148326 (PMC4740490; doi:10.1371/journal.pone.0148326)

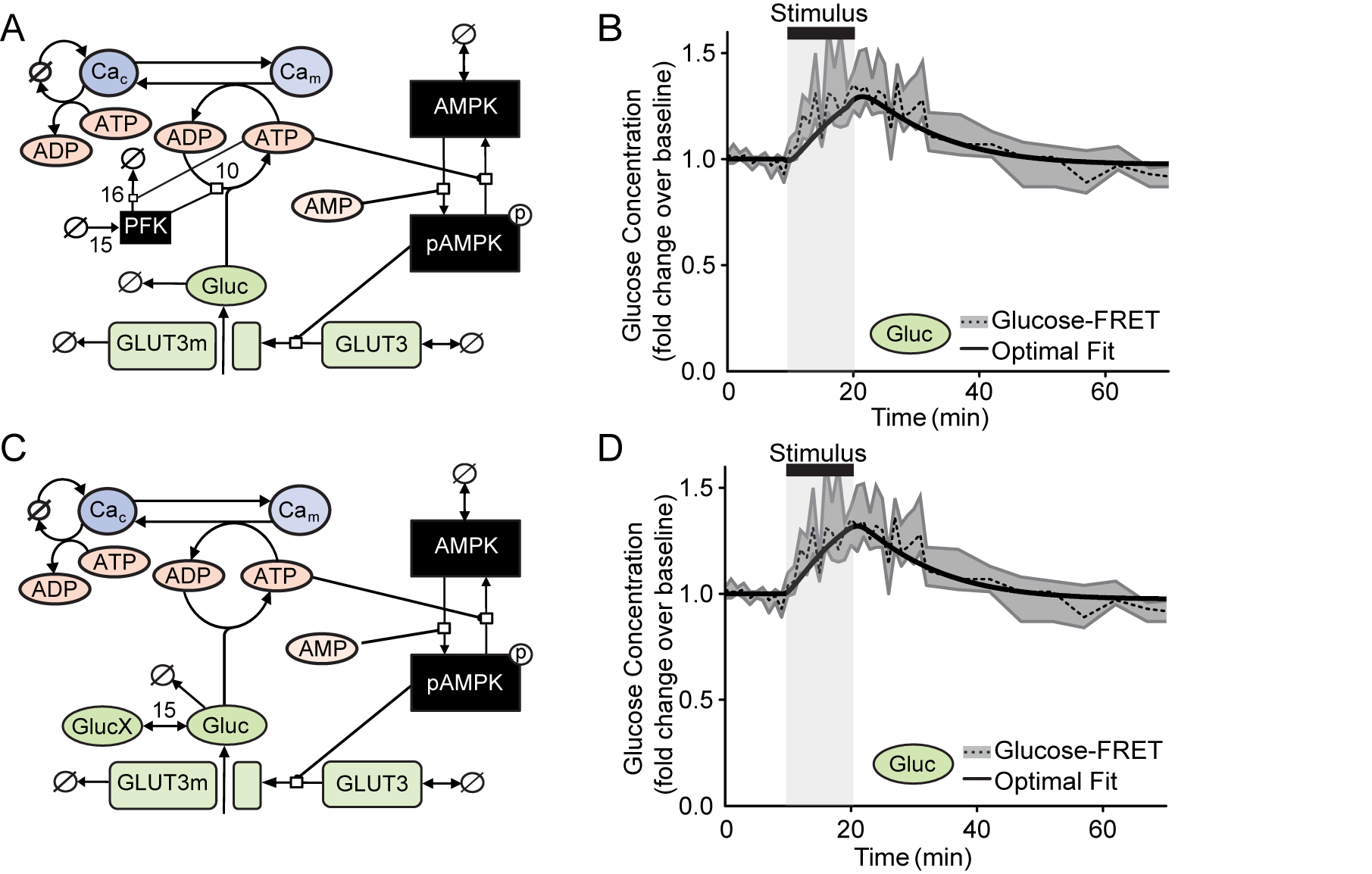

Supplement: S1 Fig — (A) Model extension 1 additionally incorporated enzymatic (phosphofructokinase, PFK) regulation of ATP production (Rx 10) via ATP-mediated negative feedback (Rx 16) but (B) model performance was not improved over the original model, as illustrated by the predicted glucose dynamics (black line), overlaid on single-cell fluorescence measurements from [15]. (C) Model extension 2 implemented a reversible glucose store (Rx 15), as may be provided by the endoplasmic reticulum or via glycogen metabolism, but (D) did not alter model performance. (TIF) [file pone.0148326.s001.tif]
