# Supplementary material for: Computational Analysis of AMPK-Mediated Neuroprotection Suggests Acute Excitotoxic Bioenergetics and Glucose Dynamics Are Regulated by a Minimal Set of Critical Reactions
Source: PLoS One. 2016 Feb 3;11(2):e0148326. doi: 10.1371/journal.pone.0148326 (PMC4740490; doi:10.1371/journal.pone.0148326)
Supplement: S1 Table — Numbered flux expressions align with reactions in Fig 1A and Table 2. The differential equation for cytosolic calcium (Cac) includes the ca_mag term applied as model input. The input was applied between t > ca_onset and t < ca_onset + ca_duration. Ca_onset and ca_duration were set to 10 min, and were varied around this value to simulate fluctuations in cellular response. (PDF) [file pone.0148326.s002.pdf]

<u>Table S1:</u> Model flux expressions, differential equations and stoichiometric matrix

Flux expressions:

**j1**	$k_{on}1$
**j1a**	$k_{on}1a * Ca_c * ATP$
**j2**	$k_{on}2$
**j2a**	$k_{off}2 * AMPK$
**j3**	$k_{on}3$
**j3a**	$k_{off}3 * GLUT3$
**j4**	$k_{on}4 * Ca_c$
**j5**	$k_{on}5 * Ca_m * ATP^{0.8}$
**j6**	$k_{on}6 * AMP * AMPK$
**j7**	$k_{on}7 * pAMPK * GLUT3$
**j8**	$k_{off}8 * GLUT3_m$
**j9**	$k_{on}9 * GLUT3_m$
**j10**	$k_{on}10 * Gluc * ADP$
**j11**	$k_{on}11 * ADP * ADP$
**j11a**	$k_{off}11 * ATP * AMP$
**j12**	$k_{on}12 * ATP * pAMPK$
**j13**	$k_{off}13 * Gluc$

Differential Equations:

$$d\frac{[Ca_c\ ]}{dt} = j1 - j1a - j4 + j5 + \ \mathrm{ca_{mag}}.[(\mathrm{t} > \mathrm{ca_{onset}}\ \ \&\mathrm{t} < (\mathrm{ca_{onset}} + \mathrm{ca_{duration}})]$$

$$\frac{d[Ca_m]}{dt} = j4 - j5$$

$$\frac{d[AMPK]}{dt} = j2 - j2a - j6 + j12$$

$$\frac{d[pAMPK]}{dt} = j6 - j12$$

$$\frac{d[GLUT3]}{dt} = j3 - j3a - j7$$

$$\frac{d[GLUT3_m]}{dt} = j7 - j8$$

$$\frac{d[Gluc]}{dt} = 25 * j9 - j10 - j13$$

$$\frac{d[ATP]}{dt} = -j1a - 0.8 * j5 + j10 + j11 - j11a$$

$$\frac{d[ADP]}{dt} = j1a + 0.8 * j5 - j10 - 2 * j11 + 2 * j11a$$

$$\frac{d[AMP]}{dt} = j11 - j11a$$

$$\frac{d[AMPKAR]}{dt} = 0.17 * \frac{d[pAMPK]}{dt}$$ (does not contribute to network stoichiometry)

Stoichiometric Matrix

	**j1**	**j1a**	**j2**	**j2a**	**j3**	**j3a**	**j4**	**j5**	**j6**	**j7**	**j8**	**j9**	**j10**	**j11**	**j11a**	**j12**	**j13**
Ca_c	1	-1					-1	1									
Ca_m							1	-1									
AMPK			1	-1					-1							1	
pAMPK									1							-1	
GLUT3					1	-1				-1							
$GLUT3_m$										1	-1						
Gluc												25	-1				-1
ATP		-1						-0.8					1	1	-1		
ADP		1						0.8					-1	-2	2		
AMP														1	-1		
