# Supplementary material for: Computational Analysis of AMPK-Mediated Neuroprotection Suggests Acute Excitotoxic Bioenergetics and Glucose Dynamics Are Regulated by a Minimal Set of Critical Reactions
Source: PLoS One. 2016 Feb 3;11(2):e0148326. doi: 10.1371/journal.pone.0148326 (PMC4740490; doi:10.1371/journal.pone.0148326)
Supplement: S2 Table — Parameter sets from three simulations as highlighted by the yellow, cyan and red data points in Fig 2B–2E. All metrics displayed in Fig 2B–2E were calculated for each simulation. (PDF) [file pone.0148326.s003.pdf]

Table S2: Parameter sets from Figure 2B-E

Steady-state concentrations (nM unless otherwise stated)

	Yellow	Cyan	Red
**Ca_c**	213	183	171
**Ca_m**	290	281	350
**AMPK**	160	170	131
**pAMPK**	70	66	89
**AMPKAR**	70	66	89
**GLUT3**	439	416	420
**GLUT3m**	214	221	246
**Glucose**	4.42 mM	4.72 mM	4.86 mM
**ATP**	2.77 mM	2.62 mM	2.34 mM
**ADP**	165.3 μM	231.2 μM	224.3 μM
**AMP**	24.4 μM	25.2 μM	21.9 μM

Kinetic Constants (s^{-1}, nM s^{-1} or nM^{-1} s^{-1})

	Yellow	Cyan	Red
**$k_{on}1$**	27.0	18.6	18.6
**$k_{on}1a$**	46 x 10^{-9}	39 x 10^{-9}	47 x 10^{-9}
**$k_{on}2$**	4.4 x 10^{-3}	5.5 x 10^{-3}	3.5 x 10^{-3}
**$k_{off}2$**	28 x 10^{-6}	32 x 10^{-6}	27 x 10^{-6}
**$k_{on}3$**	111 x 10^{-3}	123 x 10^{-3}	121 x 10^{-3}
**$k_{off}3$**	12.5 x 10^{-6}	14.7 x 10^{-6}	13.0 x 10^{-6}
**$k_{on}4$**	29.8	53.4	52.0
**$k_{on}5$**	153 x 10^{-6}	255 x 10^{-6}	204 x 10^{-6}
**$k_{on}6$**	1.15	0.89	1.63
**$k_{on}7$**	3.5 x 10^{-6}	4.3 x 10^{-6}	3.1 x 10^{-6}
**$k_{off}8$**	495 x 10^{-6}	527 x 10^{-6}	471 x 10^{-6}
**$k_{on}9$**	5.2 x 10^{3}	4.9 x 10^{3}	5.1 x 10^{3}
**$k_{on}10$**	279 x 10^{-12}	287 x 10^{-12}	261 x 10^{-12}
**$k_{on}11$**	93 x 10^{-9}	47 x 10^{-9}	39 x 10^{-9}
**$k_{off}11$**	37.6 x 10^{-9}	37.6 x 10^{-9}	38.4 x 10^{-9}
**$k_{on}12$**	23 x 10^{-3}	22 x 10^{-3}	22 x 10^{-3}
**$k_{off}13$**	0.25	0.23	0.26
**$k_{on}14$**	0.16	0.17	0.18

Influx Characteristics (calcium influx magnitude and duration)

	Yellow	Cyan	Red
**Ca_mag (AU)**	34.3	34.2	34.6
**Ca_duration (s)**	621	644	593
