# Supplementary material for: Computational Analysis of AMPK-Mediated Neuroprotection Suggests Acute Excitotoxic Bioenergetics and Glucose Dynamics Are Regulated by a Minimal Set of Critical Reactions
Source: PLoS One. 2016 Feb 3;11(2):e0148326. doi: 10.1371/journal.pone.0148326 (PMC4740490; doi:10.1371/journal.pone.0148326)
Supplement: S3 Table — Flux expressions, differential equations, steady-state concentrations and kinetic constants for each model variant are listed here if they differ from the model described in the main paper. See Tables 1–3 and S1 Table to compare with main model. (PDF) [file pone.0148326.s004.pdf]

Table S3: Model extensions –parameters, equations and steady-state constraints altered from original model

Refer to Tables 1-3 and S1 to compare with original model

Model Extension 1:

State Variable	Description	Steady-State Conc. (μM)
Ca_c	Cytosolic Calcium	0.15
**ATP**	Adenosine Triphosphate	2.5×10^3
**ADP**	Adenosine Diphosphate	280
**AMP**	Adenosine Monophosphate	28
**PFK**	Phosphofructokinase	1

Flux expressions:

j10 $k_{on}10 * Gluc * ADP * PFK$
j15 $k_{on}15$
j16 $k_{on}16 * ATP * PFK$

Differential Equations:

$$\frac{d[Gluc]}{dt} = 25 * j9 - j10 - j13$$

$$\frac{d[ATP]}{dt} = -j1a - 0.8 * j5 + j10 + j11 - j11a$$

$$\frac{d[ADP]}{dt} = j1a + 0.8 * j5 - j10 - 2 * j11 + 2 * j11a$$

$$\frac{d[PFK]}{dt} = j15 - j16$$

Rx #	Reaction Equation	Half-Life [min]	k_{on} [$nM.s^{-1}$] or [$nM^{-1}.s^{-1}$]	k_{off} [$nM^{-1}.s^{-1}$]	Reaction Description
**1***	$\rightarrow Ca_c$		14.4		
**4***	$Ca_c \rightarrow Ca_m$		2.6×10^3	-	
**5***	Ca_m + ATP*0.8 → Ca_c + ADP*0.8		9.7×10^{-3}	-	
**6***	AMP + AMPK → AMP + pAMPK		0.96	-	
**10**	Gluc + ADP + PFK → ATP+PFK		10×10^{-12}	-	
**11***	ADP + ADP <=> ATP + AMP		5.1×10^{-9}	6×10^{-9}	
**15***	→ PFK		82.5×10^3	-	PFK production
**16†**	PFK + ATP → ATP	350 [1]	33×10^{-6}	-	ATP-mediated PFK degradation

* k_{on} values determined from steady-state constraints

† k_{off} values determined from half-life ($k_{off} = \ln(2) / t_{1/2}$).

Parameter	Steady-State Constraint
$\boldsymbol{k_{on}4}$	$\frac{1.25(ADP_{ss}.Gluc_{ss}.PFK_{ss}.k_{on}10 - ATP_{ss}.Ca_{css}.k_{on}1a)}{Ca_{css}}$
$\boldsymbol{k_{on}5}$	$\frac{1.25(ADP_{ss}.Gluc_{ss}.PFK_{ss}.k_{on}10 - ATP_{ss}.Ca_{css}.k_{on}1a)}{ATP_{ss}^{0.8}.Ca_{mss}}$
$\boldsymbol{k_{on}9}$	$\frac{Gluc_{ss}.ADP_{ss}.PFK_{ss}.k_{on}10 + Gluc_{ss}.k_{off}13}{25.Glut3m_{ss}}$
$\boldsymbol{k_{on}15}$	$ATP_{ss}.PFK_{ss}.k_{on}16$

Model Extension 2:

State Variable	Description	Steady-State Conc. (μM)
**GlucX**	Intracellular Glucose Store	1

Flux expressions:

j15 $k_{on}15 * GlucX$
j15a $k_{off}15 * Gluc$

Differential Equations:

$$\frac{d[Gluc]}{dt} = 25 * j9 - j10 - j13 + j15 - j15a$$

$$\frac{d[GlucX]}{dt} = -j15 + j15a$$

Rx #	Reaction Equation	k_{on} [s^{-1}], [$nM.s^{-1}$] or [$nM^{-1}.s^{-1}$]	k_{off} [s^{-1}] or [$nM^{-1}.s^{-1}$]	Reaction Description
**15***	GlucX <=> Gluc	26 x 10^3	0.23	Intracellular reversible glucose store

* k_{on} values determined from steady-state constraints

Parameter	Steady-State Constraint
$\boldsymbol{k_{on}15}$	$\frac{Gluc_{ss}.k_{off}15}{GlucX_{ss}}$

References:

[1] Jones GM & Mayer RJ. Degradation of glucose-metabolizing enzymes in the rat small intestine during starvation. Biochem J 1973;132(4): 657-61
